# Complete remission of alpha-fetoprotein-producing gastric cancer by combined tislelizumab-apatinib treatment of a patient with proficient mismatch repair: a case report

**DOI:** 10.1186/s12957-022-02751-7

**Published:** 2022-09-08

**Authors:** Jinyu Xiang, Wenjing Gong, CongCong Wang, Ping Sun, Aina Liu

**Affiliations:** Departments of Oncology, The Affiliated Yantai Yuhuangding Hospital of Qingdao University, Yantai, 264000 Shandong China

**Keywords:** Alpha-fetoprotein-producing gastric cancer, Tislelizumab, Apatinib, Antiangiogenesis, Immune checkpoint inhibitor

## Abstract

**Background:**

Alpha‑fetoprotein-producing gastric cancer (AFPGC) is a rare type of gastric cancer with a high rate of metastasis and poor prognosis. Despite substantial progress in the treatment of many solid tumors, there are no reports of the safety and effectiveness of immune checkpoint inhibitors in combination with antiangiogenesis agents for AFPGC patients who have proficient mismatch repair.

**Case presentation:**

We describe a 69-year-old man who was diagnosed with metastatic AFPGC. After progression to chemotherapy resistance, tislelizumab combined with apatinib was administered, although the patient’s gastroscopic pathology showed proficient mismatch repair. After three cycles of therapy, partial remission (reduced by 56%) was obtained, and the quality of life improved significantly. Surprisingly, after more than 1 year of continuous application of the combination treatment regimen, both the primary and metastatic tumors in this patient eventually disappeared, which obtained complete remission without surgery. The patient has had a progression-free survival of more than 24 months and is still continuing to benefit.

**Conclusions:**

This case is the first example of effective treatment of AFPGC with tislelizumab combined with apatinib. The outcomes of this case suggest a highly effective and tolerable therapeutic strategy for microsatellite-stabilized AFPGC.

## Introduction

Alpha-fetoprotein (AFP)-producing gastric cancer (AFPGC) is a relatively rare disease, accounting for 1.3–15% of gastric cancer patients [1]. Alpha-fetoprotein-producing gastric cancer is usually identified as primary gastric cancer with a serum AFP concentration greater than 20 ng/ml or positive AFP immunohistochemical staining. Compared with non-AFP gastric cancer, AFPGC is associated with poor prognosis because of high rates of liver and lymph node metastases [2]. The conventional treatment for gastric cancer includes surgery, chemotherapy, targeted therapy, and immunotherapy. Immunotherapy is based mainly on biomarkers, such as the programmed cell death ligand 1 (PD-L1), microsatellite status, and tumor mutational burden (TMB). Tislelizumab is a monoclonal antibody with high affinity and specificity for PD-1. By minimizing FcɣR binding to macrophages, tislelizumab was designed to prevent antibody-dependent phagocytosis, a putative mechanism of T-cell clearance and resistance to anti-PD-1 treatment [3]. In the Rationale 205 study (NCT03469557), tislelizumab plus chemotherapy produced durable responses with manageable tolerability as first-line treatment of patients with advanced esophageal squamous cell carcinoma or gastric/gastroesophageal junction adenocarcinoma [4]. A worldwide multicenter phase 3 trial, Rationale 305 ((NCT03777657), is in progress to assess tislelizumab with chemotherapy versus placebo plus chemotherapy as first-line treatment of patients with gastric or gastroesophageal junction cancer [5].

Apatinib is a small molecule drug that exhibits significant antiangiogenic and antitumor effects [6]. The China Food and Drug Administration (CFDA) has authorized apatinib as a third-line treatment for advanced gastric cancer. For patients with progressing hepatocellular carcinoma, a trial of anti-PD-1 antibody SHR-1210 in combination with apatinib (VEGFR2 TKI) demonstrated encouraging therapeutic efficacy [7]. Low-dose apatinib improved the tumor microenvironment and boosted the antitumor effect of PD-1/PD-L1 blockade in lung cancer [8].

Combinations of immune checkpoint inhibitors and antiangiogenic drugs have synergistic effects while retaining a good safety profile [9]. Inhibition of VEGF not only causes tumor vascular normalization but also VEGF inhibition promotes tumor CD8 + T-lymphocyte infiltration and improves tumor immunotherapy [10]. Conversely, PD-1/PD-L1 inhibitors can regulate tumor blood vessels by activating effector T cells and upregulating interferon-γ production, thereby increasing the efficacy of antiangiogenic drugs and improving effector T-cell infiltration and killing [11]. Consequently, PD-1/PD-L1 inhibitors coupled with antiangiogenic medicines can create a mutually beneficial positive feedback loop.

In this report, we describe treatment of a 69-year-old man who had AFPGC with numerous metastases. The patient responded to therapy with tislelizumab plus apatinib and achieved complete remission. After twenty-two treatment cycles, follow-up imaging indicated that both the original tumor and metastatic lesions had shrunk and finally vanished. The patient’s vital signs were stable, and he had a better quality of life.

## Case presentation

**Fig. 1:**
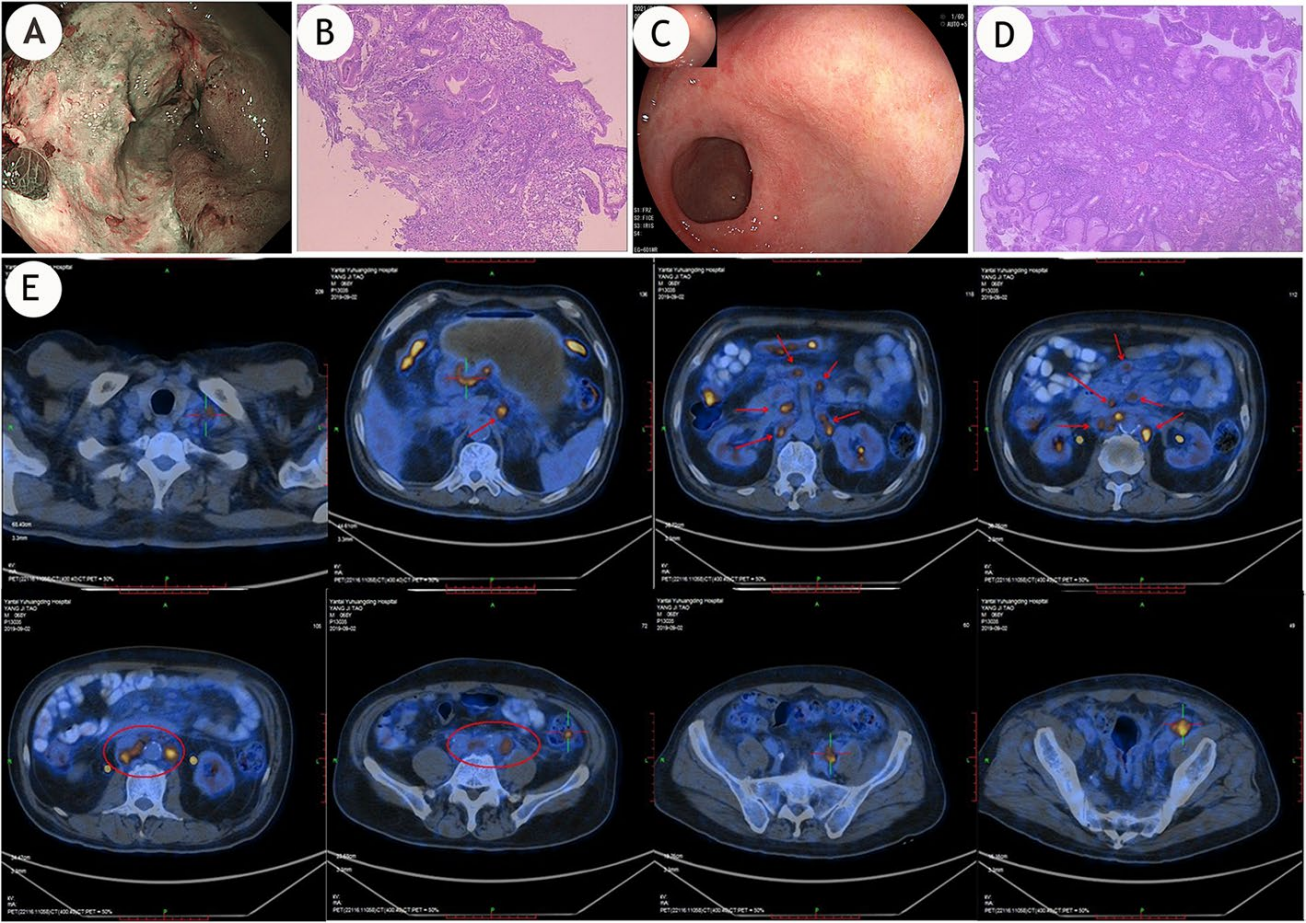
Baseline data of the patient. **A** Gastric ulcer found during gastroscopy. **B** Pathological findings (gastric antrum) adenocarcinoma, part of which showed signet ring cell carcinoma differentiation. **C** After more than a year of treatment, the gastric ulcer lesions (cancer) disappeared in April 2021. **D** The antrum mucosal tissue is inflammatory, and the glandular epithelium presents mild intestinal metaplasia. **E** PET/CT showed gastric cancer with multiple lymph node metastases in the left supraclavicular fossa, lesser omentum sac, retroperitoneal, mesenteric root, and left iliac artery

In August 2019, a 69-year-old man presented to the Yantai Yuhuangding Hospital with upper abdominal pain. He had type 2 diabetes. No family history was identified. His Eastern Cooperative Oncology Group score was 1. His hemoglobin concentration was 72 g/L, and the erythrocyte count was 2.91 × 10^12^. Serum cancer embryonic antigen was 117.2 ng/mL (normal concentration < 5.00 ng/ml), AFP was 131.6 ng/mL (normal concentration < 7.02 ng/ml), and other tumor markers were generally normal. On August 29, 2019, an enhanced abdominal and pelvic CT scan showed gastric cancer with multiple lymph node enlargement in the perigastric, mesenteric travel region, and retroperitoneal region. Endoscopy showed a gastric antrum ulcer, which was to be confirmed (Fig. 1A).

Gastroscopic pathology (Fig. 1B, August 30, 2019) demonstrated adenocarcinoma in gastric antrum, part of which showed signet ring cell carcinoma differentiation. According to serological and histopathological findings, the tumor was diagnosed as AFPGC instead of hepatoid adenocarcinoma of the stomach.

Immunohistochemistry indicated HER2 (0), Ki67 (+ 90%), MSH2 ( +), MSH6 ( +), PMS2 ( +), and MLH1 ( +). PD-L1 (22C3) combined positive score (CPS) = 3 (mainly tumor cells), positive control (DAKO0905 +), and negative control ( −).

In situ hybridization findings are as follows: Eber ( −). Four common mismatch repair gene proteins (MLH1, MSH2, MSH6, and PMS2) were detected by immunohistochemistry. If any protein is lost (negative expression), it is considered deficient mismatch repair, and if all four genes are positive, it is considered proficient mismatch repair.

PET/CT (Fig. 1E) showed nonuniform thickening of the gastric wall in the antrum area, a coarse serous surface, and increased fluorodeoxyglucose (FDG) metabolism in the thickened gastric wall, consistent with PET/CT findings of gastric cancer. Multiple enlarged lymph nodes in the left supraclavicular fossa, lesser omentum sac area, retroperitoneum, mesenteric root, and left iliac vessels, with increased FDG metabolism, and lymph node metastasis were considered.

**Fig. 2:**
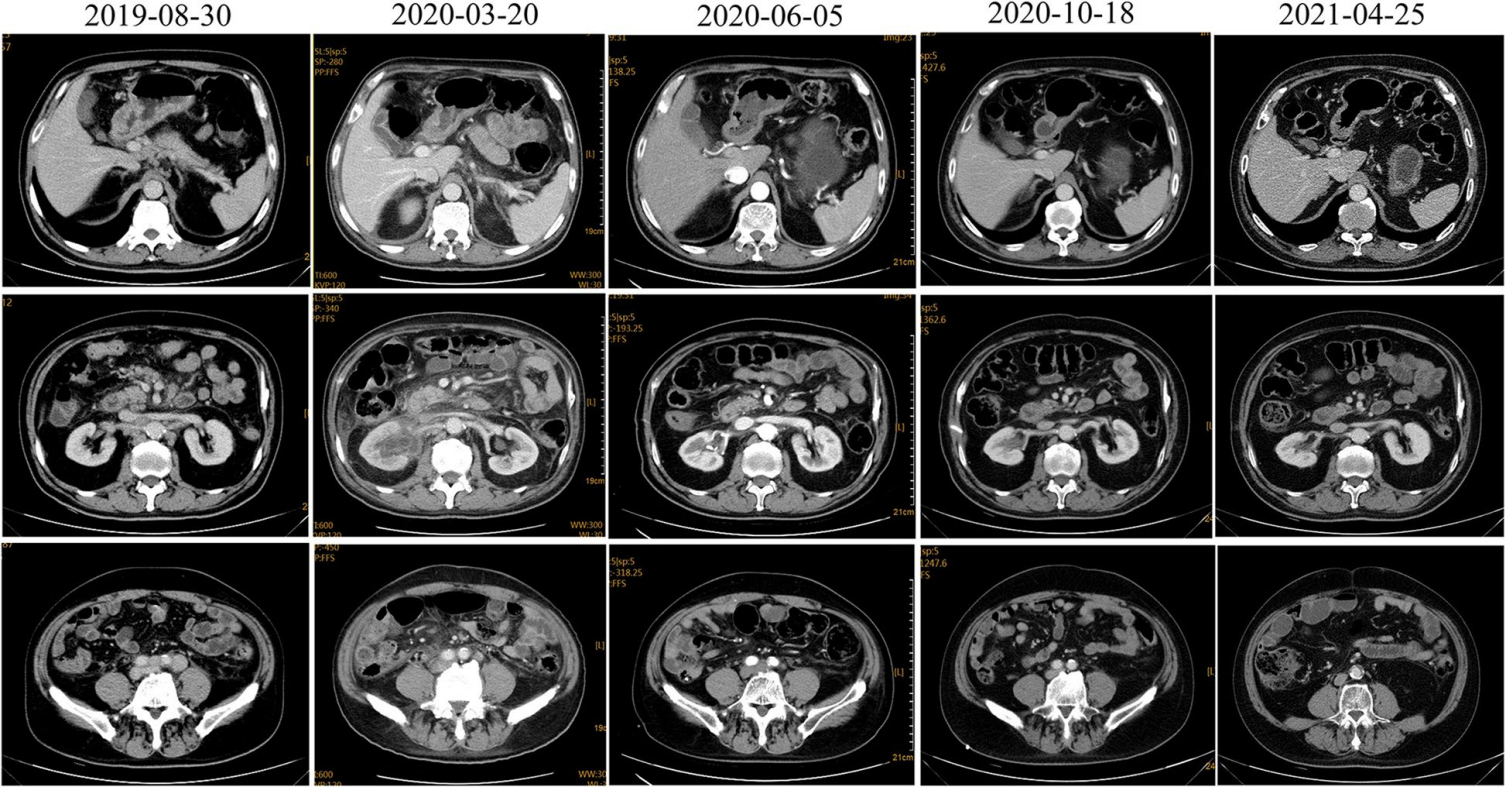
By regular CT scan, cancer in gastric antrum and metastases in other parts became progressively. Eventually, the tumor disappeared completely

**Fig. 3:**
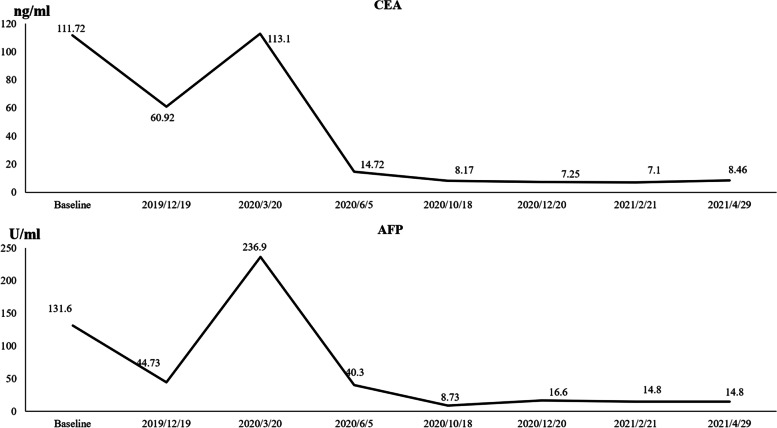
Concentrations of the tumor markers carcinoembryonic antigen (CEA) and alpha-fetoprotein (AFP)

The patient was definitively diagnosed as having AFP-producing gastric cancer (stage 4) with multiple lymph node metastases in the left supraclavicular fossa, lesser omentum sac, retroperitoneal, mesenteric root, and left iliac artery. After the anemia was improved, the hemoglobin concentration rose to 95 g/L. After four cycles of standard first-line chemotherapy with oxaliplatin (230 mg/d1) combined with S-1 (60 mg bid d1-14/21d), the disease was evaluated as stable according to the response evaluation criteria in solid tumors (RECIST 1.1). Because the efficacy was unsatisfactory, albumin paclitaxel was added in the fifth cycle in combination with oxaliplatin, and, due to anemia, treatment was stopped on the 8th day. From January 12, 2020, to February 26, 2020, the 6th, 7th, and 8th cycles of chemotherapy were administered as follows: albumin-bound paclitaxel 200 mg d1 and 8 + S-1 60 mg bid d1-14/21d. After six cycles, the lesion remained stable. However, the tumor progressed significantly after eight cycles. On March 20, 2020, re-examination of the abdominal and pelvic cavities with enhanced CT (Fig. 2) indicated that the gastric antrum wall was thicker than earlier, and multiple enlarged lymph nodes in the abdominal cavity and retroperitoneum were enlarged. There are changes in the right renal pelvis, the right upper ureter, and its surroundings, and the metastatic tumor has invaded the ureter, the right renal vein, and the inferior vena cava. There was hydronephrosis in the right kidney, hydrops around the ureter in the renal sinus and upper segment, hydrops around the testicular sheath, and subcutaneous soft tissue edema in the abdominal pelvis wall; these findings suggested progressive disease. The concentrations of serum cancer embryonic antigen and AFP increased to 113.1 ng/ml and 236.9 ng/ml, respectively, after eight cycles (Fig. 3). On March 23, 2020, due to urinary tract obstruction caused by the compression of the metastatic tumor, a right nephrostomy was performed under local anesthesia for the patient. Because of the rapid disease progression and limited efficacy of chemotherapy, tislelizumab (once every 3 weeks) plus oral apatinib (250 mg once daily) was administered in our hospital beginning on March 31, 2020.

After three cycles of treatment, CT examination (Fig. 2, June 5, 2020, vs. March 20, 2020) revealed that the thickening of the gastric antrum wall was thinner than earlier, and the multiple enlarged lymph nodes in the abdominal cavity and retroperitoneal area were reduced. The metastatic tumor of the right renal pelvis and the upper part of the right ureter was smaller, peripheral exudation and effusion were better than before, the right renal vein and inferior vena cava were clearer, and pelvic effusion and testicular hydrocele were basically absorbed. Bladder wall thickening and abdominal pelvis wall edema were better than before. The carcinoembryonic antigen decreased to 8.17 ng/ml, and AFP decreased to 8.73 ng/ml (Fig. 3). After three cycles, the tumor shrank by 56%, and the efficacy evaluation showed a large partial response. In December 2020, the patient’s right hydronephrosis had completely disappeared, and the right nephrostomy was removed. At review of the patient, at the end of April 2021, surprisingly, endoscopy showed that the gastric tumor and intraperitoneal metastasis had disappeared, and that the patient had achieved complete remission (Fig. 1 C and D, Fig. 2). The patient achieved 24 months of progression-free survival with the combination treatment by now, which significantly improved the quality of life. During treatment, the patient experienced grade 2 treatment-related hypertension according to the National Cancer Institute Common Terminology Criteria for Adverse Events version 5.0; this hypertension was relieved after symptomatic treatment. The patient has taken nifedipine, a calcium channel blocker for his hypertension. The patient said that he does not take it every day and only uses it when his blood pressure is high. Moreover, his blood pressure has not elevated since November 2021, and he has stopped taking this antihypertensive medication. As of the last follow-up visit, on April 3, 2022, the patient was continuing with this combined treatment.

## Discussion

The gastric cancer of the patient described in this report progressed after first-line standard chemotherapy, and the metastatic tumor caused obstruction of the right urinary tract. The rapid progression of the disease reflects the grade malignancy of AFPGC. The patient finally achieved complete remission with tislelizumab plus apatinib treatment following conventional chemotherapy. At present, progression-free survival is more than 24 months, and the patient is well.

AFPGC is defined as gastric cancer with elevated serum AFP > 20 ng/mL. AFPGC has high proliferative activity, weak apoptotic activity, and rich neovascularization compared with AFP-negative gastric cancer [12]. AFPGC responds poorly to chemotherapy, and basic research has indicated that AFP-producing cell lines are less sensitive to many drugs, including platinum and fluoropyrimidines [13]. However, Wang et al. reported that the triplet regimen as first-line chemotherapy improved the prognosis of AFPGC accompanied by liver metastasis [14]. Li et al. and Wang et al. reported that apatinib had a good effect on AFPGC [15, 16], which is consistent with a case report by Arakawa et al. on the efficacy of antiangiogenic therapy of AFPGC [17]. However, apatinib alone has limited efficacy in treatment of AFPGC. The median progression-free survival of patients administered apatinib was 3.5 months (95% *CI*: 2.34–4.66). The median overall survival was 4.5 months (95% *CI*: 3.49–5.51) [15]. Li et al. suggested that PD-1 checkpoint inhibitors plus chemotherapy could be beneficial for AFPGC [18]. AFPGC patients respond to immunotherapy, possibly due to their specific genetic features. Arora et al. [19] suggested that TCGA gastric adenocarcinomas with elevated expression of AFP demonstrated aggressive behavior and showed inferior survival, independent of grade and stage, and a distinct genetic profile. Pathology findings for the patient in this case showed proficient mismatch repair. The microsatellite was stable, or microsatellite instability was low, but the PD-L1 expression level by CPS was 3. In accordance with the NCCN and CSCO guidelines, the patient should have been treated with apatinib, monoimmunotherapy, or other drugs. In the REGONIVO study [20], regorafenib in combination with nivolumab showed promising results (*ORR* 44%, median PFS 5.8 months) in the third-line or subsequent treatment of a gastric cancer cohort with microsatellite stability. The objective response rate was 60.0% for patients with PD-L1 *CPS* ≥ 1, and the median progression-free survival was 10.9 months. In addition, the EPOC1706 study [21] on ASCO-GI (Gastrointestinal Cancers Symposium) of lenvatinib plus pembrolizumab for advanced gastric cancer showed an objective response rate of 69% and a median progression-free survival of 7.1 months. The above suggests that immunotherapy combined with antiangiogenic tyrosine kinase inhibitor therapy is indeed more effective than monotherapy. We hypothesized that immune checkpoint inhibition combined with antiangiogenesis treatment had a better effect on the case of AFPGC described in our report.

Because antiangiogenic and immune checkpoint inhibitors both target the tumor microenvironment, a combination of immune checkpoint inhibition with antiangiogenic drugs might have a synergistic antitumor effect [22]. Blocking VEGF and the PD-1/PD-L1 axis is beneficial in a variety of cancers, and this approach is developing as a desirable combination therapy. Immune checkpoint inhibitors have been demonstrated to be complementary to antiangiogenic treatment in many preclinical and clinical investigations. Conversely, antiangiogenesis reduces the expression of several immunological checkpoints and increases the percentage of antitumor/protumor immune cells, therefore blocking inhibitory immune signals. Immune checkpoint inhibitor treatment can restore the microenvironment of immune support and promote vascular normalization, which is conducive to drug delivery and reducing the dosage of immune checkpoint inhibitors and the risk of adverse events [23]. Treatment with VEGFR thymidine kinase inhibitors in combination with immune checkpoint inhibitors has improved outcomes in RCC, HCC, and NSCLC; mucosal melanoma, endometrial carcinoma, esophageal carcinoma, triple-negative breast cancer, microsatellite stability GC, and CRC; and head and neck squamous cell carcinoma, urothelial carcinoma, osteosarcoma, and other malignant tumors [24]. The combination therapies of anti-PD-1/PD-L1 treatments with antiangiogenic agents in the REGONIVO study demonstrated activation of immune checkpoints that resulted in more potent antitumor activity compared with anti-PD-1 monotherapy [7, 8]. Thus, additional work is needed to identify the mechanism for the modulation of PD-L1 expression via antiangiogenesis in AFPGC.

## Conclusion

For AFPGC patients, a combination of antiangiogenic therapy with an immune checkpoint inhibitor appears to be an efficacious approach to treating this type of tumor. According to the available preclinical and clinical evidence, a combination of an immune checkpoint inhibitor with a drug that targets VEGF may enhance the overall result. We report that a patient with AFPGC achieved durable complete remission from the combined treatment of the PD-1 inhibitor tislelizumab and the antiangiogenic agent apatinib with mild toxicity. We suggest that an antiangiogenic agent combined with immunotherapy will be a promising treatment for patients with AFPGC. The regimen is worth further investigation in clinical trials.

## Data Availability

All data generated or analyzed during this study are included in this published article.

## Abbreviations

AFP: Alpha-fetoprotein
GC: Gastric cancer
AFPGC: Alpha-fetoprotein-producing gastric cancer
ICI: Immune checkpoint inhibitor
pMMR: Proficient mismatch repair
MSS: Microsatellite stability
TME: Tumor microenvironment
VEGF: Vascular endothelial growth factor
PD-1: Programmed cell death 1
RCC: Renal cell carcinoma
HCC: Hepatocellular carcinoma
CRC: Colorectal cancer
NSCLC: Non-small cell lung cancer
PD-L1: Programmed cell death ligand 1
CR: Complete remission
ECOG: Eastern Cooperative Oncology Group
CEA: Cancer embryonic antigen
CPS: Combined positive score
